# Parametric Study of Biodiesel Synthesis from Waste Cooking Oil Using Ca-Rich Mixed Metal Oxides

**DOI:** 10.1021/acsomega.5c00916

**Published:** 2025-04-23

**Authors:** Emine Emel Çakırca, Ayşe Nilgün Akın

**Affiliations:** Faculty of Engineering, Chemical Engineering Department, Kocaeli University, 41380 Kocaeli, Turkey

## Abstract

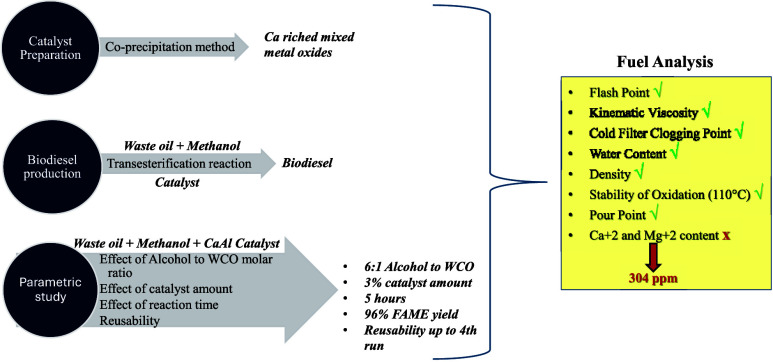

Biodiesel production from waste cooking oil (WCO) is a key step in sustainable energy production. The use of heterogeneous catalysts in the biodiesel production process offers significant advantages, particularly due to their reusability and low environmental impact. In this study, calcium-containing mixed metal oxide catalysts, derived from Ca-rich hydrotalcite-like structures and prepared at various metal oxide ratios, were evaluated for their catalytic activity in the transesterification of WCO with methanol. Parametric studies were conducted to investigate the effects of alcohol-to-oil ratio, catalyst amount, and reaction time on the performance of the most active catalyst, CaAl hydrotalcite-like. The fatty acid methyl ester (FAME) yield was calculated by using the EN14103 method. The optimal reaction conditions were determined to be 338 K for 5 h, a methanol-to-WCO molar ratio of 6:1, and a 3% catalyst amount. Under these conditions, a FAME yield of 96% was achieved using CaAl hydrotalcite as the catalyst. The reusability of the CaAl hydrotalcite-like catalyst was also assessed in the transesterification of WCO, showing no significant loss of activity after the first four uses. However, further reusability tests revealed that the CaAl hydrotalcite-like catalyst dissolved in the reaction medium over time. Fuel tests on the obtained biodiesel indicated that it met the requirements of the European Biodiesel Standard EN 14214.

## 1 Introduction

Energy is a critical component of sustainable socioeconomic development. It is widely accepted that the provision of affordable and reliable energy sources is a fundamental requirement for the economic growth and stability of a nation. Currently, the majority of the energy is derived from petrochemical sources. However, since these resources are finite, renewable and highly efficient energy sources have gained increasing importance. Consequently, research on energy derived from biomass has steadily growing.

Biodiesel is a liquid biofuel, consisting of a mixture of fatty acid alkyl esters, obtained from the transesterification of oil with alcohol in the presence of a catalyst. It has significant potential to replace traditional diesel fuel because it is environmentally friendly, biodegradable, renewable, and can be used in internal combustion engines without modifications.^1,2^ However, the production cost of biodiesel is higher than that of diesel fuel derived from fossil sources. One effective way to reduce this cost is to use high free fatty acids or nonedible oils as raw materials.^3^ Waste cooking oils (WCOs) represent a promising raw material for biodiesel production and can significantly reduce the production costs. Moreover, the disposal of WCO poses serious environmental challenges by polluting water and soil. Utilizing WCO as a raw material for biodiesel production, rather than disposal, is one of the most effective ways to mitigate these environmental impacts.^4,5^ Despite these advantages, the oil quality must be improved. The presence of water in the oil leads to the hydrolysis of triglycerides, increasing the free fatty acid content. These free fatty acids then undergo a saponification reaction in an alkaline medium, which decreases the biodiesel yield.^6^ For this reason, the free fatty acid content of waste oils should be kept below 1% in order to effectively use basic catalysts in the transesterification reaction.^7^ Several strategies for improving biodiesel production from waste oil include reducing free fatty acid content, using acid catalysts, and applying high pressure and temperature.^8^ In a study by Sahar Sadaf et al., WCO was filtered to remove insoluble impurities and heated to 100 °C to remove moisture. After purification, WCO was pretreated with an acidic esterification process to reduce the free fatty acid content, making the oil suitable for transesterification.^9^ Similar pretreatment processes for waste oils prior to biodiesel production are commonly reported in the literature.^10−14^

**Figure 1:**
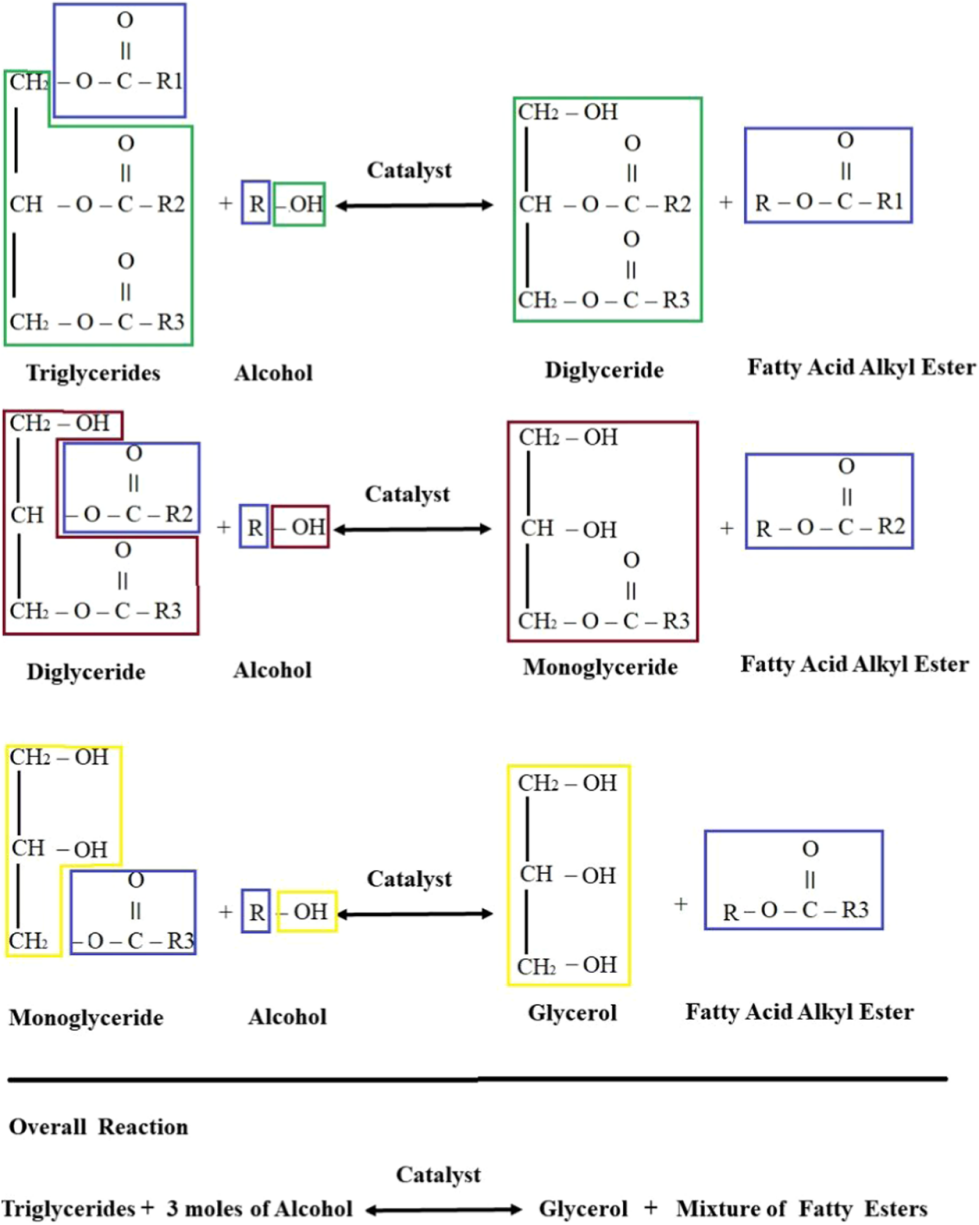
Mechanism of the transesterification reaction of triglycerides (adapted with permission from Santos et al., Energies, MDPI, 2019).^16^

The transesterification process is described as the reaction of triglycerides and short-chain alcohols in the presence of a catalyst. This process consists of three reversible reaction mechanisms, where triglycerides are converted into diglycerides, diglycerides into monoglycerides, and monoglycerides into glycerol. In each step, the glyceride formed reacts with a short-chain alcohol to form fatty acid methyl ester.^15^ The mechanism of the reactions is shown in Figure 1.

The transesterification reaction is known to have a slow reaction rate. To increase both the reaction rate and yield, a catalyst is necessary.^17,18^ Among heterogeneous catalysts, CaO, MgO, SrO, and BaO are the most widely studied due to their high activity. However, the solubility of SrO and BaO catalysts in the reaction medium is high, resulting in poor reusability.^19^ In 1984, Peterson demonstrated the high activity of CaO-based catalysts in the transesterification reaction in his graduate study, and since then, CaO catalysts have been investigated in subsequent studies.^20^ In the literature, CaO catalysts are used in pure, mixed, or supported forms.^12,21−25^

Kesic et al.^26^ explained the transesterification reaction mechanism over the CaO catalyst in detail. The transesterification reaction begins over the CaO catalyst with the attack of a methoxide ion on the carbonyl carbon of a triglyceride molecule bound to the catalyst surface, forming a tetrahedral intermediate. In the second step, the unstable tetrahedral intermediate is rearranged, and the diglyceride anion and the fatty acid methyl ester are dissociated. The diglyceride anion is then stabilized by a proton from the catalyst surface to form the diglyceride, and at the same time, the active site on the catalyst surface is regenerated. The methoxide anion then attacks another carbonyl carbon atom in the diglyceride, forming another mole of methyl ester and monoglyceride. These three steps are repeated until all three carbonyl centers of the triglyceride are attacked by methoxide ions, yielding one mole of glycerol and three moles of methyl ester.^26^

Aghel et al.^12^ reported the production of biodiesel from WCO with methanol using MgO/CaO catalysts. In this study, the transesterification reaction was carried out with an oil-to-methanol volumetric ratio of 2:1, a catalyst amount of 9%, a reaction temperature of 63 °C, and a reaction time of 2 h. Under these conditions, they achieved a FAME yield of 93%.^12^ A study by Alsaiari et al.^27^ used eggshells as a natural CaO source, utilizing waste palm oil as the feedstock. The study achieved approximately 85% FAME yield with 4% catalyst, a 12:1 ethanol-to-oil molar ratio, 75 °C, and 3 h of reaction time. Additionally, when the reusability of the CaO catalyst was examined, the FAME yield decreased to 71% after the third use under the same reaction conditions, attributed to the accumulation of organic contaminants on the catalyst surface.^27^ Another recent study by Widayat et al.^28^ investigated mixed metal oxides, specifically CaO/MgO/Fe_3_O_4_ catalysts, for the transesterification of WCO and methanol. The study achieved an approximately 96% conversion.^28^ In another study, Ghasemi et al.^29^ examined a CaO–ZrO_2_ nanocatalyst supported on acid-treated kaolinite for WCO esterification and transesterification. The reaction conditions were 110 °C for 3 h with a 10% catalyst, resulting in a total conversion (esterification and transesterification) of 93%. The catalyst demonstrated negligible performance degradation after the fifth use.^29^ Santos et al.^16^ used eggshell, a natural waste, as a source of CaO catalyst to perform the transesterification of soybean oil. The eggshell, in the form of CaCO_3_, was calcined at 850 °C for 3 h to convert it into CaO. A FAME conversion of approximately 86% was achieved using an amount of 5% catalyst and a methanol-to-oil ratio of 12:1.^16^

Hydrotalcite-like structures are highly preferred as catalytic materials in the transesterification reaction due to their versatility in chemical composition. Gao et al.^30^ studied biodiesel production from palm oil by loading KF onto a CaAl hydrotalcite catalyst. When KF, which has high basicity, was loaded, the biodiesel yield increased to 97% under the reaction conditions of a methanol/oil molar ratio of 12:1, a 5% catalyst by weight, a reaction time of 5 h, and a temperature of 110 °C. Nowicki et al.^31^ synthesized ZrMgAl hydrotalcites using the coprecipitation method and achieved a 99% oil conversion under the reaction conditions of 120 °C, 6 h of reaction time, 5 atm pressure, and high alcohol/oil molar ratios. It was observed that Zr loaded onto the hydrotalcite-like catalyst structure strengthened the basic properties of the catalyst. A similar study was also conducted by Wang and Jehng.^32^ They synthesized hydrotalcite-like catalysts by replacing Mg^2+^ ions with Ni^2+^, obtained MgAlNi16 catalyst, and achieved FAME yield of 87%. In the literature, there are many studies on hydrotalcite catalysts, similar to those mentioned above. However, it is observed that very few studies have been conducted on hydrotalcite catalysts prepared by utilizing the basic properties of Ca^2+^.

In our previous study, Ca-containing hydrotalcite-like catalysts were designed and synthesized based on the basic strength of Ca^2+^ and the stability of the hydrotalcite catalyst, and we investigated their catalytic activities in the transesterification of microalgae oil with methanol. The transesterification reaction was conducted under the following conditions: 65 °C for 5 h, an oil/methanol molar ratio of 1:6, and a 3 wt % catalyst, yielding up to 90% FAME with the CaMgAl_2_ catalyst. The study highlighted the significant influence of the amount and structure of Ca in the catalyst on the FAME yield, and it was seen that high conversion is achieved with CaMgAl hydrotalcite-like catalysts without any support material.^33^

Building on this previous work, the current study investigates the use of these Ca-containing hydrotalcite-like catalysts for biodiesel production from WCO. The reaction parameters and the properties of the obtained biodiesel are analyzed in detail. To determine the optimal reaction conditions, the most active catalyst was employed, and its reusability was thoroughly assessed. The leaching behavior of the catalyst was examined and interpreted by using ICP, FTIR, and XRD techniques. Finally, the conformity of the obtained biodiesel with the ASTM D6751 and EN 14214 standards was evaluated. This study is original in the literature due to the use of WCO as a raw material and the application of Ca-rich hydrotalcite-like structures as catalysts in the transesterification process.

## 2 Experimental Section

### 2.1 Materials

The catalysts were prepared using these chemicals: Al(NO_3_)_3_ 9H_2_O (95%, Sigma-Aldrich), Ca(NO_3_)_2_ 4H_2_O (99%, Merck), Mg(NO_3_)_2_ 6H_2_O (97%, Sigma-Aldrich), NaOH (98%, Merck), and Na_2_CO_3_ (99,9%, Merck). For the transesterification reaction, methanol (99%, Merck) and WCO were used. *n*-Hexane (98.5%, Merck) and methyl heptadecanoate (99%, Sigma-Aldrich) were used for the GC analysis of the obtained biodiesel.

### 2.2 Methods

The catalysts used in this study were prepared via coprecipitation method, and *x* value (*x* = Al^3+^/Al^3+^ + Ca^2+^ + Mg^2+^ molar ratio) was adjusted to be 0.33 according to the hydrotalcite structure and was labeled to refer to a Ca/Mg molar ratio as MgAl, CaAl, CaMgAl0.5, CaMgAl1, and CaMgAl2. The synthesis process and its characterizations were described in our previous study.^33^ WCO used in the transesterification reaction was obtained from a private catering company in the Gebze district in Turkey. The fatty acid content and acidity value of the used WCO are given in Table 1.

**Table 1:** Fatty Acid Distribution and Acidity Value of the WCO

fatty acid name	fatty acid content, wt, %	acidity, mg KOH/g oil
C16 (palmitic acid)	9	0.76
C18:1 (oleic acid)	38	
C18:2 (linoleic acid)	49	
C18:3 (linolenic acid)	4	

The transesterification reactions of WCO with methanol, in the presence of catalysts, were conducted in a reaction system consisting of a 250–1000 mL three-necked glass reactor equipped with a magnetic stirrer and a Heidolph brand heater. A water-cooled condenser was integrated into the reactor to prevent evaporated methanol from escaping the system during the reaction. The reaction was carried out at a constant stirring speed of 700 rpm and a reaction temperature of 65 °C. Upon completion of the reaction, the catalyst was filtered and removed from the mixture and the resulting product was centrifuged to separate the biodiesel phase. The transesterification reaction parameters for all of the catalysts are presented in Table 2.

**Table 2:** Transesterification Reaction Parameters and Conditions of the WCO

the catalysts	reaction temperature, °C	molar ratio of alcohol/oil	catalyst amount, % weight of oil	reaction time, h	stirring speed, rpm
CaAl	65	3/1, 6/1, 9/1, 12/1, 15/1	1, 3, 5, 7, 9	1, 2, 3, 4, 5, 6	700
MgAl	65	6/1	3	5	700
CaMgAl0.5	65	6/1	3	5	700
CaMgAl1	65	6/1	3	5	700
CaMgAl2	65	6/1	3	5	700

FAME yield of all of the catalysts was examined under mild reaction conditions to determine the most active catalyst. The parametric study was carried out over this catalyst to determine the optimum reaction conditions of the transesterification of WCO with methanol.

The obtained biodiesel was placed in a 1 mL Eppendorf tube and centrifuged for 10 min at 10,000 rpm. After centrifugation, the biodiesel yield was analyzed in terms of the FAME yield using an Agilent 6890 gas chromatograph according to the EN14103 method. Fuel analysis of biodiesel was conducted according to the EN 14214 and ASTM D6751 standards.

The reusability of the CaAl catalyst in the transesterification of methanol with waste oil was investigated over six cycles at 65 °C with a reaction time of 5 h, a 6:1 alcohol-to-oil molar ratio, and a 5% catalyst loading. At the end of each reaction, the catalyst was filtered and added to the next reaction mixture without further treatment. Conversion was measured for each cycle. To analyze changes in the catalyst used, a new experimental procedure was applied. The catalysts from the first, second, third, fourth, fifth, and sixth cycles were filtered, washed with hexane and methanol, and dried. These processes served as preliminary preparations for characterization tests to observe changes in the catalyst structure after the reaction. The change in the FAME content was analyzed using a GC/FID system, while the biodiesel structure was examined by the FTIR analysis. The metal concentration in biodiesel was analyzed according to the EN 14538 method. Catalyst characterization at the end of each cycle was performed by using XRD, FTIR, and ICP/OES techniques.

## 3 Results

### 3.1 Effect of the Catalysts on the Activity of WCO Transesterification Reaction

**Figure 2:**
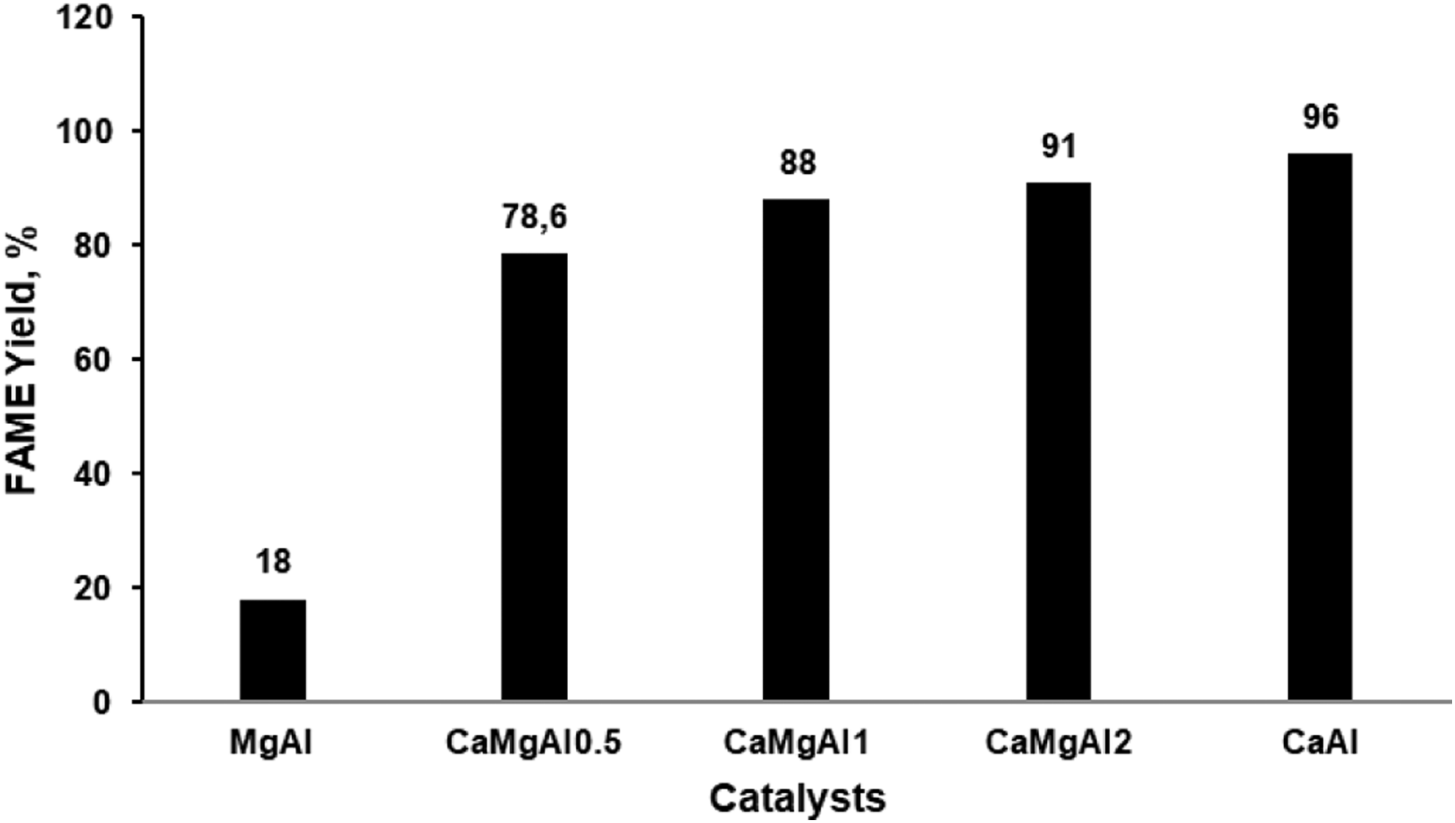
Effect of different catalysts on the transesterification of WCO with methanol.

To investigate the effect of CaO loading on the catalytic activities of catalysts, a series of Ca-containing mixed hydrotalcite-like catalysts were prepared at different Ca/Mg ratios and tested in the transesterification of waste cooking oil (WCO) with methanol. The transesterification was carried out under mild conditions: 65 °C for 5 h, with an oil-to-methanol molar ratio of 1:6, and a catalyst loading of 3% by weight. The aim of this study is to identify the most active catalyst under the optimal reaction conditions, similar to those used in the literature where the highest conversion is achieved.^30,32−37^ The catalytic activities of the catalysts are shown in Figure 2.

The activities of the catalysts were listed as CaAl > CaMgAl2 > CaMgAl1 > CaMgAl0.5 > MgAl, and it was observed that the FAME efficiency for Ca-containing catalysts is over 75%. It has been observed that adding Ca to MgAl catalysts with a Ca/Mg ratio of 0.5 significantly increases the biodiesel yield. When the rate of Ca addition increased to 2 units, the biodiesel yield increased to 90% or more. The most active catalyst was CaAl with a 96% yield of FAME. In this catalyst, the ratio of the concentration of Al^3+^ metal ions to the total concentration of metal ions (*x* = Al^3+^/Al^3+^ + Ca^2+^) is 0.33, and the greater amount of Ca^2+^ ions has led to an increase in CaO forms. Additionally, the presence of strong basic sites and the large pore size in the CaAl catalyst have enhanced the yield of the transesterification reaction. From the results, the effect of the Ca content and catalyst structure on activity is more important than the Mg content in the catalyst. The homogeneous distribution of active metal oxide forms on the surface, large crystal structures and pore sizes, and high basicity in Ca-containing catalysts positively affected the catalyst activity.^33^ Although reactions using CaAl hydrotalcite-like catalysts are not common in the literature, it has been observed that studies on CaO catalysts in the production of biodiesel from waste oil have increased recently.^14,23,27−29^ Xu et al.^38^ designed three-dimensional Mg–Ca–Al hydrotalcite-like catalysts with a Ca/Mg ratio of 1 and modified the obtained catalysts with KF in a ratio of 1:1 (KF:HTLCs). Under optimum reaction conditions, a 90% conversion was achieved from these catalysts.^38^ Guzmán-Vargas et al.^36^ obtained a 90% yield of Jatropa oil into biodiesel after a 4 h reaction with a Ca/Mg molar ratio of 1 and a 30% KF loading. Similarly, Xu et al.^39^ synthesized the CaMgAl hydrotalcite catalyst on an inorganic ceramic and tested its activity in the transesterification of palm oil with methanol. They achieved a 17% conversion and increased the activity of the catalyst to 92% by loading 76% KF into the catalyst.^39^ Compared to findings in the literature, the CaMgAl hydrotalcite-like catalysts prepared in this study show high activity in waste oil transesterification without adding any additives (such as KF, KOH).

### 3.2 Effect of Reaction Conditions on the Activity of CaAl Catalyst

#### 3.2.1 Effect of Reaction Time

**Figure 3:**
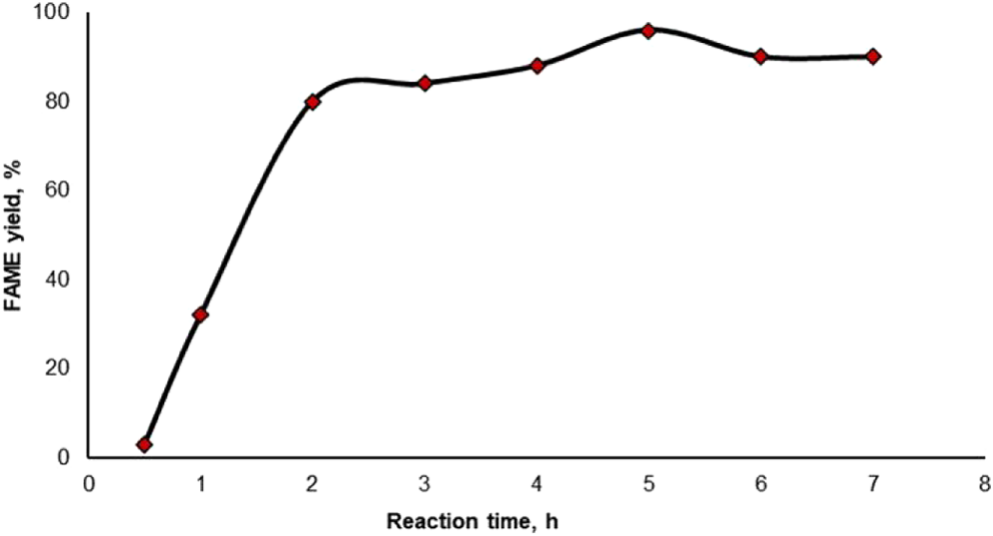
Effect of reaction time on the transesterification of WCO with methanol over the CaAl catalyst.

Figure 3 shows the effect of the reaction time on the FAME yield over the CaAl catalyst. The reactions were conducted at 65 °C with a 3% catalyst concentration and a waste-oil-to-methanol molar ratio of 1:6 and were continued for up to 7 h. Product analysis was performed using the GC/FID system, with samples taken every hour. Initially, there was almost no fatty acid methyl ester (FAME) yield during the first half hour. However, after 30 min, the yield increased rapidly, reaching 81% at the 2 h mark. It was observed that the yield continued to increase, reaching 96% between the second and fifth hours. After the fifth hour, an increase in reaction time resulted in a decrease in the FAME yield. This reduction is attributed to the reversibility of the reaction, which caused a shift in direction and a slight decrease in biodiesel yield. Based on the results, the optimum reaction time for the studied conditions was determined to be 5 h.

#### 3.2.2 Effect of Alcohol to WCO Molar Ratio

**Figure 4:**
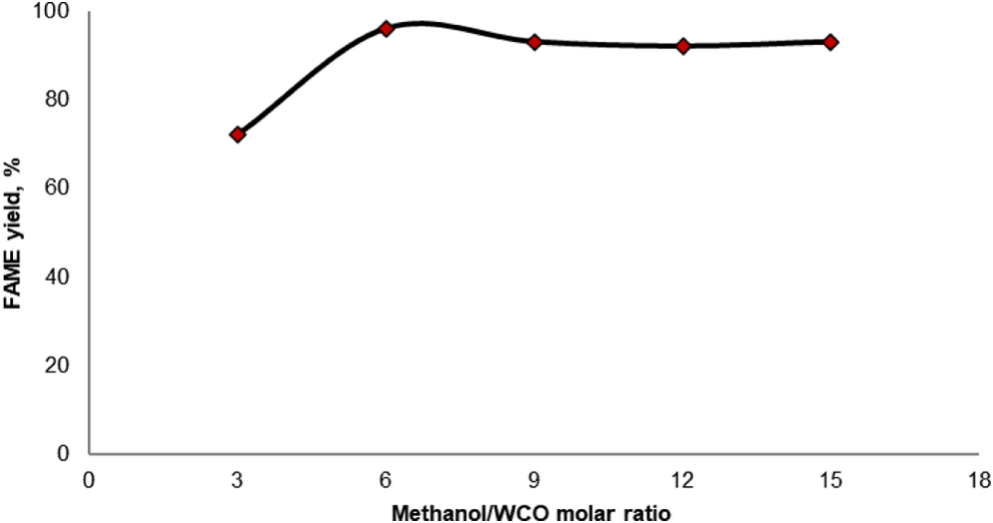
Effect of the methanol-to-WCO molar ratio on the transesterification of WCO with methanol over the CaAl catalyst.

The alcohol-to-oil ratio plays a crucial role in determining the equilibrium point of the transesterification reaction, helping to mitigate the negative effects of the reverse reaction.^40,41^ Due to the reversible nature of this reaction, a high biodiesel yield in the forward reaction is achieved either by using an excess of alcohol or by removing one of the products from the reaction mixture. In this study, experiments were initially conducted with a stoichiometric methanol-to-waste-oil ratio of 3:1. Subsequent experiments were performed with molar ratios of 6:1, 9:1, 12:1, and 15:1. The reaction conditions were kept constant at 65 °C for 5 h, with a catalyst concentration of 3%. The results are presented in Figure 4.

When the reaction was conducted with a stoichiometric ratio, the FAME content was found to be 72% at the end of the reaction. When the methanol/oil ratio was increased to 6:1, the FAME content increased to 96%. However, as the methanol/oil molar ratio was further increased, a slight decrease in the FAME content was observed, with the yield decreasing to 91%. Similar decreases have been reported in the literature, despite the use of different triglyceride sources and catalysts.^42−44^ One of the main reasons for this decrease is the lower concentration of waste oil and catalysts, as the volume of reactants increases.

#### 3.2.3 Effect of the Catalyst Amount

The effect of the catalyst amount on FAME yield was examined at catalyst concentrations of 1, 3, 5, and 9%, relative to the amount of waste oil. The reaction conditions were set at a 6:1 methanol/oil molar ratio, with a 5 h reaction time at 65 °C.

**Figure 5:**
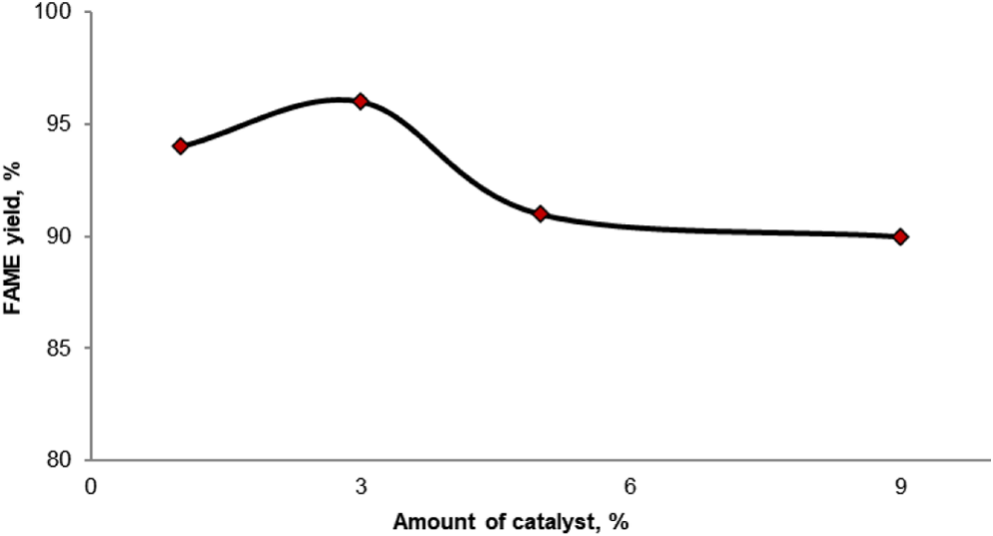
Effect of catalyst amount on the transesterification of WCO with methanol over the CaAl catalysts.

Figure 5 shows the effect of the catalyst amount on the FAME yield. When a 1% catalyst was added to the reaction, approximately 93% efficiency was obtained after 5 h. Increasing the catalyst amount to 3% resulted in a FAME yield of up to 96%. However, with further increases to 5 and 9%, FAME yields of 91 and 90% were obtained, respectively. From these results, it can be concluded that, although an increase in the catalyst amount enhances the number of basic sites, it does not significantly improve the reaction efficiency. Furthermore, as the amount of catalyst increases, resistance to mixing in the multiphase system formed prevents proper catalyst distribution. It is believed that as the reaction mixture became more viscous, poor diffusion of the reactants in the methanol-oil-catalyst system occurred, leading to a decrease in methyl ester conversion. Similar results have been reported in the literature.^43,45^

#### 3.2.4 Reusability of the CaAl Hydrotalcite-like Catalyst

**Figure 6:**
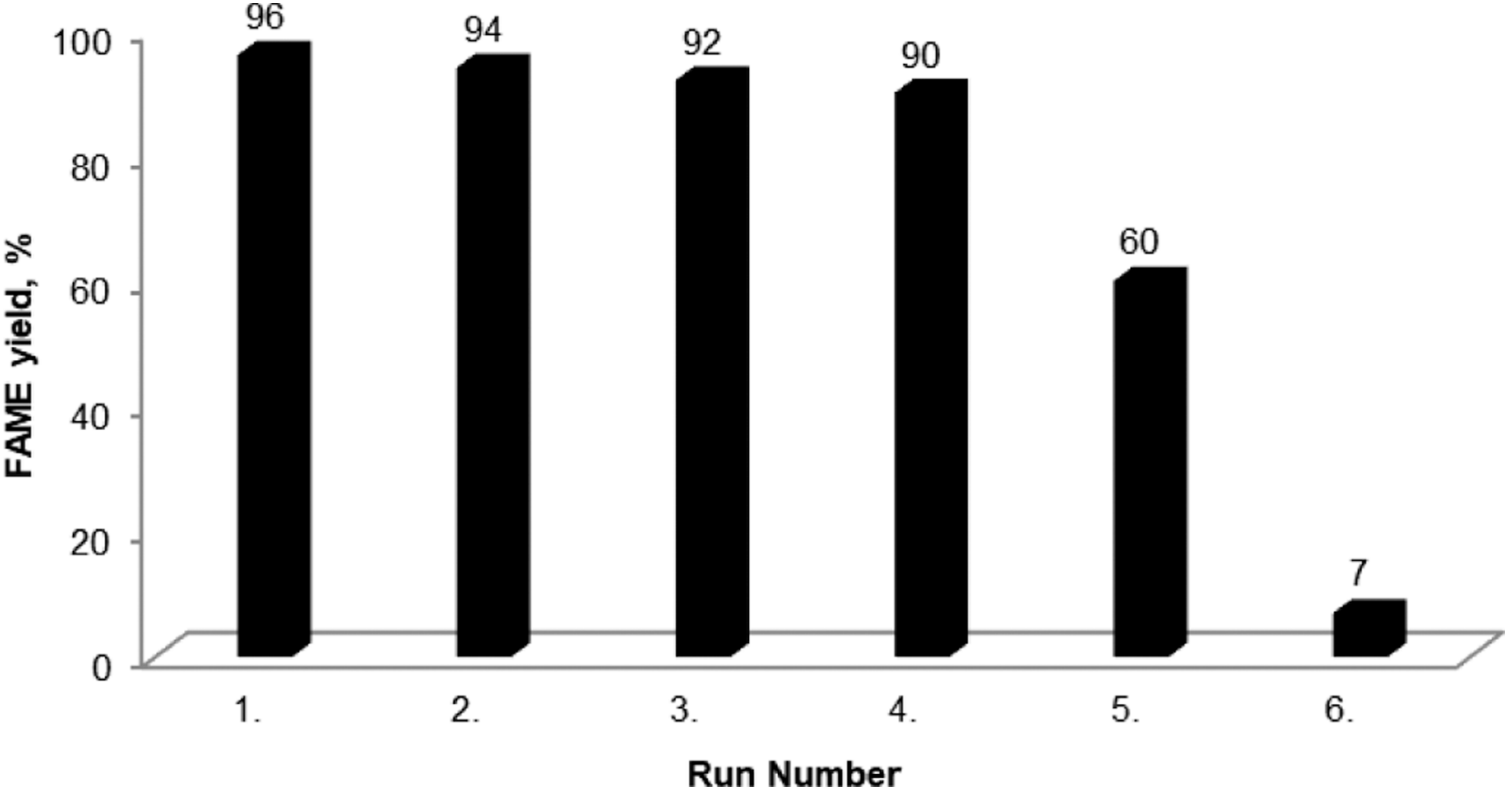
Reusability of the CaAl catalyst.

The reusability of the CaAl catalyst in the transesterification of methanol with waste cooking oil (WCO) was examined over six cycles at 65 °C with a 5 h reaction time, a 6:1 methanol/oil molar ratio, and a 5% catalyst concentration (Figure 6). At the end of each reaction, the catalyst was filtered from the reaction mixture and reused in the next cycle without further treatment. The conversions were measured for each cycle. To analyze changes in the used catalyst after each cycle, a new experimental plan was implemented. The catalysts from the first, second, third, fourth, fifth, and sixth cycles were filtered, washed with hexane and methanol, and dried. These processes served as preliminary preparations for characterization tests to observe structural changes in the catalyst after reuse.

Reusability studies showed a slight decrease in catalyst activity during the first four cycles, with the FAME content dropping from 96 to 92%. However, a significant decrease of up to 60% was observed after the fifth use. By the sixth cycle, the FAME yield decreased to approximately 10%. In this cycle, it was observed that the catalyst no longer functioned homogeneously. The primary reason for this decline is the dissolution of the catalyst in the reaction medium. The ICP/OES analysis of the catalyst revealed substantial metal loss. Similar findings have been reported in the literature.^46−49^

Determining the metal content in the obtained biodiesel is crucial for assessing catalyst inactivity and the resulting decrease in the biodiesel yield. Additionally, for biodiesel to comply with the EN 14214 European Biodiesel Standards, the metal concentration in biodiesel must not exceed certain limits. The metal content obtained from the reusability study of the catalyst is presented in Table 3.

**Table 3:** Metal Content of Biodiesels Obtained in the Reusability Study

	first run	second run	third run	fourth run	fifth run
Ca^2+^ content, ppm	302	954	1162	1539	1857
Al^3+^ content, ppm	1.98	3.83	7.68	5.2	6.34

The Ca^2+^ concentration in biodiesel is limited to a maximum of 5 ppm according to the EN 14214 standard. The Ca^2+^ content in the obtained biodiesel, however, exceeds this limit. Colombo et al. and Taufiq-Yap et al. emphasized that the high calcium content in their biodiesel was due to the presence of Ca^2+^ ions from the Ca-based catalysts they employed.^50,51^ Granados et al. examined the solubility of Ca^2+^ in methanol, methanol–glycerin, and methanol–glycerin–biodiesel mixtures and found it to be soluble in each medium. However, they noted that it dissolved primarily in glycerin.^52^

In this study, it was observed that the catalyst dissolved in the reaction medium. To better understand this phenomenon, an ICP/OES analysis was conducted on each of the catalysts used in the reusability study. This analysis allowed for determination of the extent of metal loss in the catalyst after each cycle.

As shown in Table 4, nearly 50% of Ca^2+^ was lost from the catalyst after the first use. A significant decrease was observed on the fifth use, with the Ca^2+^ content dropping from 48 to 5%. Although the Al^3+^ content showed only a slight decrease during the first reaction, it decreased by 2.6% after the fifth use, resulting in a remaining concentration of 82%. The findings from the reusability studies indicate that the catalyst surface contains impurities and its surface properties change after each use. The metal losses observed on the catalyst surface, along with the metal content in the obtained biodiesel, clearly demonstrate that the Ca^2+^ ions from the catalyst dissolved and mixed with the biodiesel.

**Table 4:** Mass Change of Ca^2+^ and Al^3+^ Metals Contained in the Catalyst as a Result of Reuse

	pure CaAl	first run	second run	third run	fourth run	fifth run
Ca^2+^ content, %	48	23.4	19.8	14.2	12.1	5.2
Ca^2+^ content, ppm	480,000	234,000	198,000	142,000	121,000	52,000
Al^3+^ content, %	14.3	14.0	10.0	4.7	3.4	2.6
Al^3+^ content, ppm	143,000	140,000	100,000	47,000	34,000	26,000

To examine the changes in the catalyst surface after reuse of the CaAl catalyst, FTIR and XRD analyses were performed. For this purpose, the catalysts used in the first three cycles were investigated.

**Figure 7:**
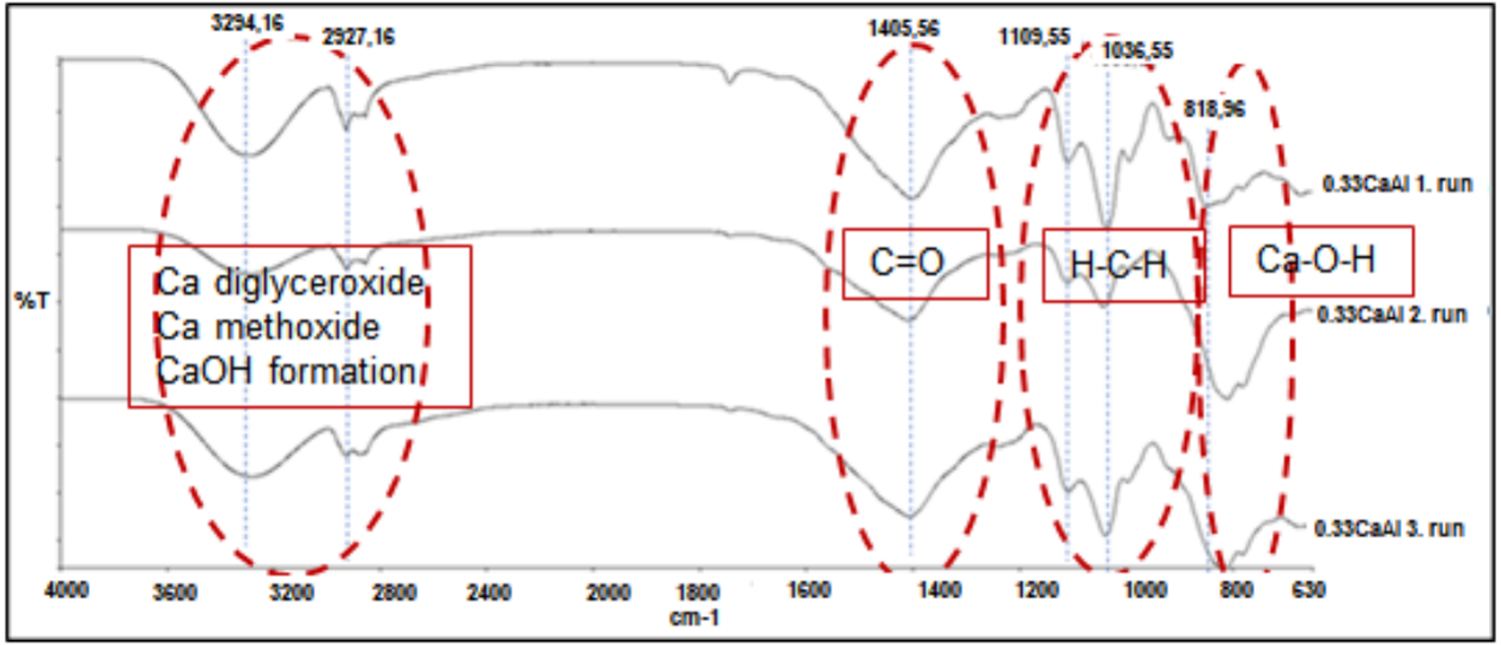
FTIR graphs of the CaAl catalyst after reuse.

Impurities were observed in the catalysts after filtration, washing, and drying, as indicated by the FTIR analysis. The FTIR spectrum shown in Figure 7 reveals that the formation of Ca diglyceride occurs in the bands between 3600 and 2800 cm^–1^. Specifically, the bands at 3294.16 and 2927.16 cm^–1^ correspond to the OH groups adsorbed on the CaO surface and the characteristic C–H stretching of Ca methoxide, respectively. This suggests that Ca methoxide is formed on the Ca diglyceride. Thaoklua et al. also noted the formation of Ca diglyceride and Ca methoxide in similar band ranges.^53^ The FTIR spectra from the reusability studies show that the catalyst surface contains impurities and that its surface properties change after each use. The unchanged catalytic activity in the first three cycles is attributed to the active nature of the formed Ca methoxide and Ca diglyceride structures during the transesterification reaction.

**Figure 8:**
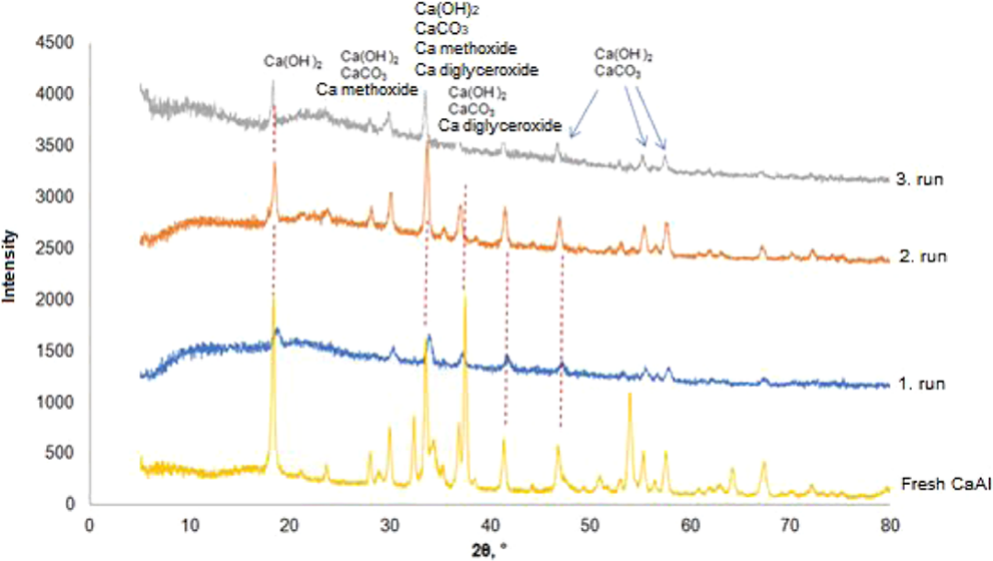
XRD pattern of the CaAl catalyst after reuse.

To better understand the phase distribution on the catalyst surface, the same catalysts were subjected to XRD analysis. The XRD pattern of the fresh CaAl catalyst was thoroughly examined in our previous study. The main peaks of the fresh CaAl catalyst are associated with the CaO and mayenite phases. The changes in the phases present in the catalyst after reaction are clearly shown in Figure 8. When the XRD results are interpreted in conjunction with FTIR analysis, the formation of Ca(OH)_2_, CaCO_3_, Ca methoxide, and Ca diglyceride is expected. The characteristic peaks of the Ca diglyceride phase appear at 2θ = 8.2, 10.2, 21.2, 24.4, 26.6, 34.4, and 36.2°.^54^ Ca methoxide shows peaks at 10.62, 21.32, 28.58, and 32.24°.^55^ The decrease in the intensity of the main peaks and small shifts in the 2θ values suggest that additional phases may have formed.

**Figure 9:**
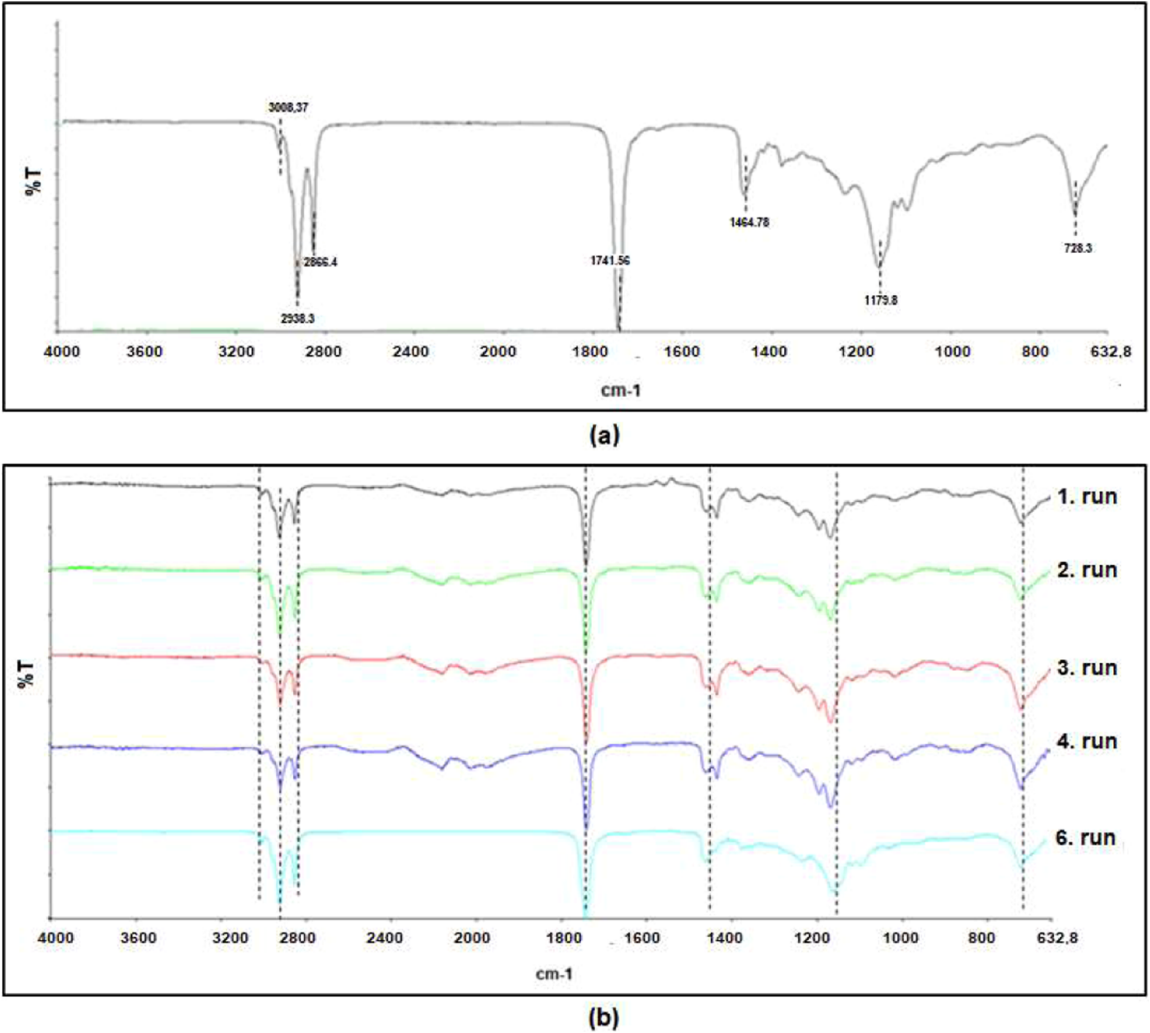
(a) Waste cooking oil FTIR image. (b) Waste oil biodiesel FTIR image.

Figure 9 shows that WCO and WCO biodiesel exhibit similar peaks due to the presence of comparable functional groups. WCO displays a single peak at 1464.78 cm^–1^, while the FTIR spectra of biodiesels obtained up to the sixth cycle in the repeatability study show two peaks at 1438 and 1461 cm^–1^. In the sixth cycle, these two peaks merge into a single peak at 1463 cm^–1^. Additionally, WCO has a single peak at 1179 cm^–1^ in the 1000–1200 cm^–1^ range, which splits into two peaks around 1200 cm^–1^ upon conversion to biodiesel. However, in the sixth cycle, a single peak formed at 1178.8 cm^–1^. The similarity of the FTIR spectrum in the sixth cycle to that of WCO indicates that the conversion of triglycerides to FAME has not been fully achieved. This also explains the reduction in the FAME content to 10% after the sixth use of the catalyst. Similar results have been reported in studies by Roschat et al.^56^ and Fereidooni and Mehrpooya.^57^ All of these findings support the notion of metal exchange on the catalyst surface and the metal content present in the biodiesel during the catalyst’s reusability cycles.

### 3.3 Fuel Analysis of Obtained Biodiesel

Biodiesel obtained through the transesterification reaction must meet certain standards to be suitable for commercial use. The quality of biodiesel is influenced by several important factors, including the composition of the feedstock, the oil extraction process, the biodiesel synthesis methodology, and the purification processes. Table 5 shows the compliance of the obtained biodiesel with standards and includes the ASTM D6751 and EN 14214 standards for biodiesel fuels and the ASTM D975 standard for petroleum diesel fuel, which was referenced from Sakthivel et al.^58^ To analyze the biodiesel fuel, the transesterification of WCO with methanol was carried out using the most active catalyst in a specially designed 1 L, three-necked glass reactor equipped with an integrated mechanical mixer. The reaction was conducted using 200 g of WCO. Methanol was added to the system to achieve a 6:1 alcohol-to-oil molar ratio. The catalyst was added in an amount corresponding to 3% by weight of 200 g of oil. The reactions were carried out at 65 °C for 5 h, aiming to produce a total of 500 mL of biodiesel. Four sets of reactions were performed, yielding approximately 650 mL of biodiesel. The obtained biodiesel was purified by filtration and centrifugation.

**Table 5:** Comparison of the Obtained Biodiesel with ASTM D6751, EN 14214, and ASTM D975 Standards^58^

property	unit	ASTM D6751	EN 14214	ASTM D975 diesel	WCO biodiesel
flash point	°C	min 130	min 101	60–80	196
kinematic viscosity	mm^2^/s	1.9–6	3.5–5	2–4.5	4.768
cold filter clogging point	°C	max +5		–8	–5
water content	mg/kg	volume % 0.05	500		381
density	kg/m^3^	880	860–900	820–860	884.7
stability of oxidation, (110 °C)	h	min 3 min	min 6 min		19 min
pour point	°C	–15 to –16		–35 to –15	–10
Ca^2+^ and Mg^2+^ contents	ppm	max 5	max 5		304

The fuel analysis of the obtained biodiesel revealed that the FAME yield was 96%. Although the alkaline metal content was 304 ppm, the density, water content, flash point, viscosity, pour point, and cold filter plugging point values complied with the ASTM D6751 and EN 14214 standards.

## 4 Conclusions

In this study, CaO-containing mixed metal oxide catalysts, derived from Ca-rich hydrotalcite-like structures, were prepared via the coprecipitation method. The catalytic activities of these catalysts were evaluated in the transesterification reaction of WCO with methanol. Among the prepared catalysts, the most active was the CaAl catalyst, achieving a 96% conversion at 65 °C for 5 h, using a methanol-to-WCO molar ratio of 6:1 and a catalyst amount of 3%. When the reusability of the CaAl catalyst was examined, a slight decrease in the FAME yield was observed up to the fourth use. Upon analyzing the metal concentrations of the used catalyst and the obtained biodiesel, it was observed that there was a significant decrease in the Ca content in the catalyst, while high amounts of Ca were present in the obtained biodiesel. The Ca content in biodiesel, reaching a concentration of 340 ppm, poses a disadvantage to the quality of the fuel.

The fuel analysis of biodiesel obtained from WCO using the CaAl catalyst indicated that all parameters were within the EN 14214 standards, except for the metal concentration. The incomplete removal of the catalyst from the reaction medium and the dissolution of Ca metal into biodiesel negatively affected its quality. Although the CaO catalyst exhibited high activity, its dissolution in the reaction medium presents a significant obstacle to its commercial application. Literature reports suggest that Ca-containing catalysts tend to leach into the reaction medium. To sustain biodiesel production using Ca-containing catalysts, which offer high activity and meet biodiesel standards, further improvements in the catalysts are necessary.
